# *BCL2* Expression at Post-Induction and Complete Remission Impact Outcome in Acute Myeloid Leukemia

**DOI:** 10.3390/diagnostics10121048

**Published:** 2020-12-04

**Authors:** Cristina Bilbao-Sieyro, Carlos Rodríguez-Medina, Yanira Florido, Ruth Stuckey, María Nieves Sáez, Santiago Sánchez-Sosa, Jesús María González Martín, Guillermo Santana, Elena González-Pérez, Naylén Cruz-Cruz, Rosa Fernández, Teresa Molero Labarta, María Teresa Gomez-Casares

**Affiliations:** 1 Molecular Biology Group, Hematology Department, Hospital Universitario de Gran Canaria Dr. Negrín, 35019 Las Palmas de Gran Canaria, Spain; hematocritico@yahoo.es (C.R.-M.); floryyana@hotmail.com (Y.F.); rstuckey@funcanis.es (R.S.); marysnow@telefonica.net (M.N.S.); jsanchez@fciisc.es (S.S.-S.); gsansan.2@hotmail.com (G.S.); gpelena88@gmail.com (E.G.-P.); nelyan@hotmail.com (N.C.-C.); tmollab@gobiernodecanarias.org (T.M.L.); mgomcasf@gobiernodecanarias.org (M.T.G.-C.); 2 Morphology Department, Universidad de Las Palmas de Gran Canaria, 35001 Las Palmas de Gran Canaria, Spain; 3 Unidad de Investigación, Hospital Universitario de Gran Canaria Dr. Negrín, 35019 Las Palmas de Gran Canaria, Spain; josu.estadistica@gmail.com; 4 Hematology Department, Complejo Hospitalario Universitario Insular Materno Infantil, 35016 Las Palmas de Gran Canaria, Spain; rfermarc@hotmail.com; 5 Medical Science Department, Universidad de Las Palmas de Gran Canaria, 35001 Las Palmas de Gran Canaria, Spain

**Keywords:** acute myeloid leukemia, BCL2 inhibitors, biomarkers, patient outcome, induction therapy, molecular diagnostics

## Abstract

Advances in acute myeloid leukemia (AML) genomics and targeted therapies include the recently approved BCL2 inhibitor venetoclax. The association between *BCL2* expression and patient outcome was analyzed in a series of 176 consecutive AML patients at diagnosis (Dx), post-induction (PI), complete remission (CR) and relapse (RL). Levels increased significantly at relapse (mean 1.07 PI/0.96 CR vs. 2.17 RL, *p* = 0.05/*p* = 0.03). In multivariate analysis, high *BCL2*-Dx were marginally associated with worse progression-free survival, while high PI levels or at CR had an independent negative impact on outcome (PI: HR 1.58, *p* = 0.014; CR: HR 1.96, *p* = 0.008). This behavior of high PI or CR *BCL2* levels and increased risk was maintained in a homogeneous patient subgroup of age <70 and intermediate cytogenetic risk (PI: HR 2.44, *p* = 0.037; CR: HR 2.71, *p* = 0.049). Finally, for this subgroup, high *BCL2* at relapse indicated worse overall survival (OS, HR 1.15, *p* = 0.05). In conclusion, high *BCL2* levels PI or at CR had an independent negative impact on patient outcome. Therefore, *BCL2* expression is a dynamic marker that may be useful during AML patient follow up, and *BCL2* levels at PI and/or CR may influence response to anti-BCL2 therapy.

## 1. Introduction

Advances in acute myeloid leukemia (AML) genomics have revealed the broad biological heterogeneity of the disease, leading to new risk stratifications in the pathology and the incorporation of targeted therapies for a more personalized management [1,2,3]. Despite this progress in the understanding of AML pathogenesis, frontline induction therapy has not substantially changed in 40 years [4] for most of the patients, and 85% of patients will relapse within 2 to 3 years [5]. BCL2 inhibitors are promising new agents [6,7] and venetoclax was approved by the U.S. Food and Drug Administration (FDA) for the treatment of AML patients under specific indications in November 2018. However, the molecular characteristics of patients likely to respond to venetoclax remain to be determined.

To address this issue, we studied the influence of *BCL2* bone marrow expression on patient outcome in a consecutive series of 176 AML patients at diagnosis (Dx), and when possible, at post-induction (PI, when the patient recovers blood count, between days 21–28 after induction [8]); morphological complete remission (CR) (i.e., <5% blasts in the bone marrow [8]) and at relapse (RL). This series included a subgroup of 52 patients aged <70 years and of intermediate cytogenetic risk. 

## 2. Materials and Methods

This study was approved by our center’s ethics committee (Comité Ético de Investigación Clínica, approval no. CEI_HUGCDN_565/150024, 01 October 2015). Informed consent was provided by all patients and donors. All methods were conducted in accordance with the Helsinki declaration and national research regulations. The datasets generated during and analyzed during the current study are available from the corresponding author on reasonable request.

Patients were diagnosed and treated according to the protocols PETHEMA LMA2010 and LMA2014 for patients aged ≤ 65 years or > 65 years, respectively, at the Hospital Universitario de Gran Canaria Dr. Negrín and the Complejo Hospitalario Universitario Insular Materno Infantil, Las Palmas, Spain, from January 2014 to July 2017 (see Table 1 for patient characteristics). They had a median age of 59 years (range 16–82); 18 were secondary AML (with an antecedent hematologic disorder or therapy-related) and 158 were de novo. Patients with acute promyelocytic leukemia were excluded from the analysis. 

Standard induction chemotherapy (anthracycline + cytarabine, “7+3” [1]) was applied in 94.9% of the patients. In consolidation, 51 patients (29%) underwent hematopoietic stem cell transplantation.

Of the consecutive bone marrow samples from 176 patients, we were able to analyze the *BCL2* expression of 156 samples from Dx and 86 at PI due to missing samples or poor sample quality (at PI, 56 patients were at CR and 30 had persistent disease). In total, 64 patients were studied at CR and 28 at RL. At three time points, we were able to study 23 patients at Dx, PI (18 CR and 5 with persistent disease) and RL; 20 patients at Dx, CR and RL.

Relative gene expression was determined by the 2-∆∆Ct method normalized to *ABL1* and relative to a cDNA pool from 10 healthy donors as internal calibrator. *BCL2* expression was analyzed in healthy donors separately and no significant variation was observed; therefore, cDNAs were pooled and introduced in each experiment. Primers annealed in exons 2 and 3 of the functional anti-apoptotic long transcript isoform [9] (ENST00000333681.5; Forward: 5′-GGATTGTGGCCTTCTTTGAG-3′; Reverse: 5′- ACAGTTCCACAAAGGCATCC -3ʹ). There was good correspondence between mRNA and protein levels (anti-Bcl-2 #610538, BD Transduction Laboratories; Supplementary Figure S1).

Student’s t-test for parametric and Wilcoxon signed-rank test for non-parametric data were used to compare continuous variables. The receiver operating characteristic (ROC) curve and area under the ROC (AUC) was used to dichotomize values of gene expression. Univariate and multivariate survival analyses were carried out simultaneously in the patient cohort and a subgroup of patients with homogeneous risk (aged < 70 years, intermediate cytogenetic risk) using the Cox proportional hazard model. All tests were two-tailed; *p* values < 0.05 were considered statistically significant. Analyses were performed using statistical software R (version 3.3.3; www.r-project.org/).

## 3. Results

Table 1 shows patients’ clinical, biological and therapeutic characteristics and their association with *BCL2* level, which was considered as both a continuous and dichotomous variable (with a cutoff established from the ROC curve of the prognostic performance and AUC). *BCL2* levels were lower in patients with *NPM1* mutation compared to *NPM1* wild-type (*p* = 0.052). Although patients with a higher cytogenetic and European LeukemiaNet (ELN) 2010 risk showed elevated *BCL2* levels, either as a continuous or dichotomous variable, this difference was not statistically significant. Mean *BCL2* mRNA expression at diagnosis was above the control bone marrow pool (*n* = 156, mean = 1.61 ± 1.16 SD, min = 0.02, max = 6.97). Analyzing the expression dynamics of the same patients at different time points (Supplementary Figure S2), compared to Dx, *BCL2* levels showed a marked descent at PI (*n* = 86, mean Dx 1.64 vs. PI 0.97, *p* < 0.001) and when achieving CR (*n* = 64, Dx 1.57 vs. CR 0.95, *p* < 0.001; Supplementary Table S1), but raised again at RL compared to PI (*n* = 23, RL 2.17 vs. PI 1.07, *p* = 0.05) and CR (*n* = 20, RL 2.27 vs. CR 0.96, *p* = 0.03). Levels were slightly higher at RL compared to those at Dx, but this difference was not significant. No association was found between *BCL2* levels and presenting CR or refractory disease at PI.

For univariate and multivariate survival analyses, only patients who received first-line intensive treatment (anthracycline + cytarabine, 7 + 3 schedule) were selected. 

*BCL2* level was also considered as a continuous and dichotomous variable for overall survival (OS, median follow up 28.4 months; range 0.2–147.3 months) and progression-free survival, defined as the time from diagnosis to disease progression or death from any cause (PFS, median follow up 24.6 months; range 6.1–147.2 months) (Supplementary Tables S2 and S3). 

At diagnosis, no association with OS was observed; however, *BCL2* levels above the AUC value (Supplementary Table S4) were significantly associated with worse PFS in the univariate analysis (HR 1.63, 95% CI 1–2.66, *p* = 0.05) and this behavior was marginally maintained in the multivariate analysis (HR 1.589, 95% CI 0.96–2.62, *p* = 0.07). 

**Figure 1:**
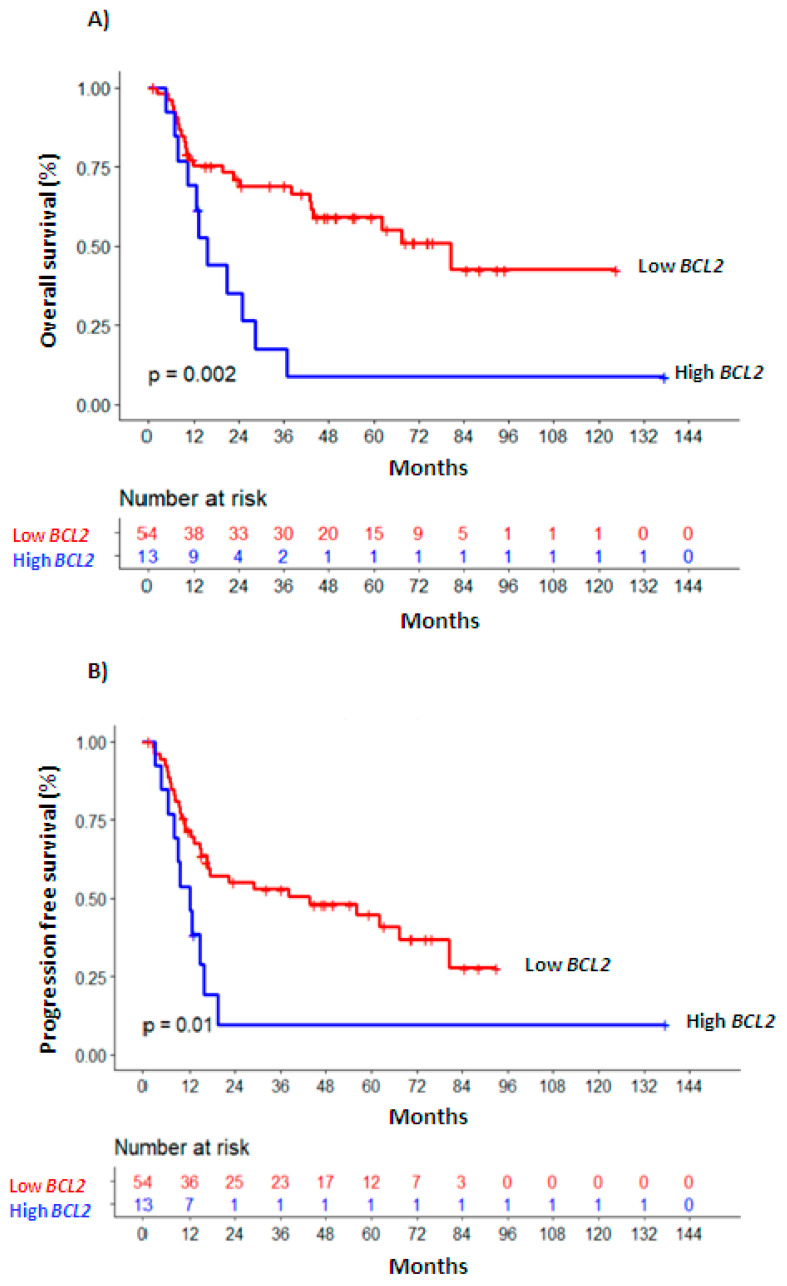
Overall survival (**A**) and progression-free survival (**B**) Kaplan—Meier curves of patients with high vs. low *BCL2* expression levels.

Univariate survival analyses were also performed considering *BCL2* levels at the moment of PI, CR (Figure 1) and at RL (Supplementary Table S5). In multivariate analysis (Table 2), higher *BCL2* levels measured at PI as a continuous variable remained as an independent marker of worse OS (HR 1.578, 95% CI 1.1–2.27, *p* = 0.014) and PFS (HR 1.547, 95% IC 1.09–2.19, *p* = 0.014); as a dichotomous variable, associated with inferior PFS (HR 1.749, 95% CI 1.04–2.95, *p* = 0.036) and marginally with OS (HR 1.764, 95% CI, 0.95–3.28, *p* = 0.073). This behavior was also maintained for OS in the subgroup of patients with homogenous risk (age < 70 years, intermediate cytogenetic risk; *n* = 52; *BCL2*-PI continuous: HR 1.63, 95% CI 1.04–2.57, *p* = 0.035; *BCL2*-PI dichotomous: HR 2.44, 95% CI 1.06–5.64; *p* = 0.037, Supplementary Table S5). 

The independent association of increased *BCL2* levels and worse OS was stronger at the time of CR (*BCL2*-CR continuous: HR 1.961, 95% CI 1.19–3.24, *p* = 0.008; *BCL2*-CR dichotomous: HR 3.028, 95% CI 1.34–6.86, *p* = 0.008, Table 2). This relationship was maintained in the homogenous subgroup, with levels of *BCL2* above the AUC value at CR (HR 2.71, 95% CI 1–7.33, *n* = 41, *p* = 0.049, Supplementary Table S5). More *BCL2* at CR was also significantly associated with worse PFS (*BCL2*-CR continuous: HR 1.73, 95% CI 1.13-2.66, *p* = 0.012; *BCL2*-CR dichotomous: HR 2.078, 95% CI 1.09–3.97, *p* = 0.027, Table 2).

Finally, we observed no significant association for the impact of *BCL2* levels on OS at the moment of RL in multivariate analyses (*BCL2*-RL continuous: *n* = 30, HR 1.105, 95% CI 0.97–1.26, *p* = 0.125). Nevertheless, in the homogenous subgroup, higher *BCL2* expression at RL was significantly associated with OS (*n* = 19, *BCL2*-RL continuous: HR 1.15, 95% CI 1–1.32, *p* = 0.05).

## 4. Discussion

We observed a positive association between the absence of *NPM1* mutation and higher *BCL2* levels. A biological explanation was not found for this association; however, recent studies show promising results for the combination of hypomethylating agents + venetoclax in older patients with a *NPM1* mutation, which could be related to the lower *BCL2* levels of these patients [10,11]. According to our results, *BCL2* expression was higher at Dx compared to PI and CR and was high again at RL; therefore, it seems to be a dynamic marker that reflects the evolution of AML and could be informative of impending RL.

Multivariate analysis of survival in relation to *BCL2* levels (as a continuous or dichotomous variable) at the different follow-up times revealed that higher *BCL2* at PI negatively influenced OS and PFS. This behavior was maintained for OS in the homogeneous subgroup. The independent association with worse OS was more evident in patients with more *BCL2* at CR in the whole series and homogenous subgroup. Lastly, albeit tested in a limited series of patients, high *BCL2* expression at RL also related with worse OS in the homogeneous subgroup. Therefore, although *BCL2* levels are generally reduced by induction chemotherapy, there is a relationship between increased *BCL2* levels at PI or when achieving CR and a worse outcome. This association remained in patients aged below 70 and of intermediate cytogenetic risk, meaning that its prognostic value may contribute to the more accurate risk stratification of this wide group of patients of variable outcome. 

Previous studies found that high BCL2 protein expression at diagnosis was associated with shorter survival in multivariate analysis [12] and this was also observed for mRNA levels in univariate analyses [13,14].

However, despite the *BCL2* reduction observed after chemotherapy, according to our results, it is the *BCL2* mRNA level after induction or at CR, rather than at diagnosis, which has the greatest influence on patient outcome. Our results reinforce the fact that the assessment of post-treatment remission by cytomorphology, defined as <5% blasts in bone marrow [8], does not mean that the patient is free of disease. It will be interesting to determine whether minimal/measurable residual disease-positive samples correlate with higher *BCL2* levels at CR (and/or PI) in future studies.

Relapsed AML cases are difficult to treat and therapeutic options are needed for refractory cases. Since sensitivity to the selective BCL2 inhibitor, venetoclax, correlates with *BCL2* levels [15], it is possible that *BCL2* expression levels at Dx, PI, CR, and even at RL may influence the response to venetoclax treatment, in agreement with our observations and recently communicated results [10,11,16,17]. 

Although further analyses in a larger series with longer follow up data are needed to confirm these results, we consider that *BCL2* expression may be an informative prognostic marker after induction therapy and could allow for the rapid, easy and cost-effective identification of a subgroup of patients in which BCL2 inhibition could be successful.

## Figures and Tables

**Table 1:** Summary of patients’ clinical, biological and therapeutic characteristics and its association with *BCL2* level.

Characteristics		*BCL2*-Dx Continuous		*BCL2*-Dx Dichotomous	
		Mean	*p* Value	High *BCL2* (%)	*p* Value
Median age at diagnosis (years)(range)	59 (16-82)		0.317		
Sex (*n* (%))					
Female	73 (41.5)		0.543	62.90	0.063
Male	103 (58.5)			37.10	
WBC per µL					
Mean (range)	6900 (900–41.2 × 10^4^)		0.304		
Cytogenetic risk (*n* (%))					
Low	13 (7.4)	1.37	0.587	58.30	0.839
Intermediate	122 (69.3)	1.59		77.70	
High	38 (21.6)	1.81		70.00	
Unknown	3 (1.7)				
ELN risk category (*n* (%))					
Low	37 (21.0)	1.39	0.345	67.60	0.283
Intermediate	66 (37.5)	1.61		76.30	
High	57 (32.4)	1.75		78.40	
Unknown	16 (9.1)				
*NPM1* (*n* (%))					
Wild type	128 (72.7)	1.7	0.052	74.60	1
Mutated	41 (23.3)	1.38		74.40	
Unknown	7 (4)				
*FLT3*-ITD (*n* (%))					
Wild type	139 (79)	1.63	0.77	73.00	0.471
Mutated	31 (17.6)	1.56		82.10	
Unknown	6 (3.4)				
Therapy (*n* (%))					
Intensive induction therapy: anthracycline + cytarabine	166 (94.9)				
Other	9 (5.1)				
Induction response (*n* (%))					
Complete remission	89 (53.9)	1.55	0.116	72.30	0.778
Primary refractory disease	66 (40)	1.81		83.10	
Exitus	10 (6)	1.4		55.60	
Relapsed disease (*n* (%))	57 (32.4)				
Allogeneic HCT in consolidation (*n* (%))					
No	91 (64.1)	1.53	0.811	73.20	0.672
Yes	51 (29)	1.58		77.30	
Exitus					
No	59 (33.5)	1.51	0.469	64.70	0.415
Yes	117 (66.5)	1.65		78.80	

Dx, diagnosis; WBC, white blood cells; ELN, European LeukemiaNet 2010; SD, standard deviation; *n*, number of cases; HCT, hematopoietic stem cell transplantation.

**Table 2:** Multivariate survival analysis.

Variable	Overall Survival				Progression Free Survival			
	*n*	HR	95% CI	*p*	*n*	HR	95% CI	*p*
	82				82			
Age (continuous)		1.024	1–1.04	0.017		1.02	1–1.04	0.029
ELN risk								
Low		1 (ref)						
Intermediate		1.139	0.54–2.41	0.733		0.773	0.39–1.52	0.454
High		1.897	0.92–3.9	0.081		1.246	0.66–2.36	0.501
*BCL2*-PI (continuous)		1.578	1.1–2.27	0.014		1.547	1.09–2.19	0.014
	82				82			
Age (continuous)		1.023	1–1.04	0.024		1.022	1–1.04	0.017
ELN risk								
Low		1 (ref)				1 (ref)		
Intermediate		1.208	0.57–2.55	0.62		0.72	0.37–1.42	0.341
High		1.896	0.92–3.9	0.082		1.262	0.66–2.39	0.477
*BCL2*-PI (dichotomous) *		1.764	0.95–3.28	0.073		1.749	1.04–2.95	0.036
	60				60			
Age (continuous)		1.032	1–1.06	0.021		1.022	1–1.04	0.017
ELN risk								
Low		1 (ref)				1 (ref)		
Intermediate		0.979	0.38–2.5	0.965		0.568	0.25–1.3	0.18
High		1.739	0.71–4.28	0.229		0.98	0.45–2.12	0.959
*BCL2*-CR (continuous)		1.961	1.19–3.24	0.008		1.732	1.13–2.66	0.012
	60				60			
Age (continuous)		1.035	1.01–1.06	0.014		1.029	1–1.06	0.021
ELN risk								
Low		1 (ref)				1 (ref)		
Intermediate		0.977	0.37–2.55	0.962		0.526	0.23–1.2	0.125
High		1.437	0.56–3.68	0.451		0.992	0.46–2.14	0.984
*BCL2*-CR (dichotomous) *		3.028	1.34–6.86	0.008		2.078	1.09–3.97	0.027

ELN, European LeukemiaNet 2010; *BCL2*-PI, *BCL2* levels at post-induction; CR, complete remission; RL, relapse; * expression above AUC value (dichotomized); HR, Hazard Ratio; CI, Confidence Interval.

## Supplementary Materials

The following are available online at https://www.mdpi.com/2075-4418/10/12/1048/s1. Supplementary Figure S1 Correspondence between protein levels (A) via Western blot and mRNA levels (B) via QRT-PCR. Protein quantification (C) by ImageJ software. The images in (A) are taken from two different blots; the samples were derived from the same experiment and the blots were processed in parallel. Supplementary Figure S2 Evolution of *BCL2* levels in 18 individual patients with follow up *BCL2* values available at diagnosis (Dx), complete remission (CR) and relapse (RL). Supplementary Table S1 Comparison of *BCL2* expression levels at different points during follow up. Supplementary Table S2 Dichotomized value of BCL2 expression level with the area under the ROC curve for overall survival (OS). Supplementary Table S3 Dichotomized value of BCL2 expression level with the area under the ROC curve for progression-free survival (PFS). Supplementary Table S4 Univariate and multivariate progression-free survival analysis in the whole series at diagnosis (Dx). Supplementary Table S5 Univariate survival analysis.
