# Supplementary material for: BCL2 Expression at Post-Induction and Complete Remission Impact Outcome in Acute Myeloid Leukemia
Source: Diagnostics (Basel). 2020 Dec 4;10(12):1048. doi: 10.3390/diagnostics10121048 (PMC7762084; doi:10.3390/diagnostics10121048)
Supplement: Supplementary file 1 [file diagnostics-10-01048-s001.pdf]

Supplementary material

Supplementary material includes tables with gene expression level information and univariate and multivariate survival analyses. It also includes a figure representing evolution of *BCL2* levels throughout follow up of patients.

Table S1. Comparison of *BCL2* expression levels at different points during follow up.

	Nº	Mean	p-value
***BCL2* Dx vs PI**	86*		
Dx		1.64	<0.001
PI		0.97	
***BCL2* Dx vs CR**	64*		
Dx		1.57	<0.001
CR		0.95	
***BCL2* PI vs RL**	23*		
PI		1.07	0.05
RL		2.17	
***BCL2* CR vs RL**	20*		
CR		0.96	0.03
RL		2.27	

Nº, number of cases; Dx, diagnosis; PI, post-induction; CR, complete remission; RL, relapse. *Of the 86 PI, 56 were at CR and 30 had persistent disease. Comparison between groups included the same patients at different time points.

Table S2. Dichotomized value of *BCL2* expression level with the area under the ROC curve for overall survival (OS)

	OS whole series					OS in <70 years and intermediate cytogenetic risk				
**Variable**	**AUC**	**95% CI**	**Cutoff**	**Sensitivity**	**Specificity**	**AUC**	**95% CI**	**Cutoff**	**Sensitivity**	**Specificity**
BCL2-DX	0.56	0.45–0.67	0.8095	0.79	0.40	0.53	0.41–0.67	2.030	0.84	0.33
BCL2-PI	0.59	0.47–0.71	1.451	0.29	0.92	0.59	0.43–0.75	1.451	0.30	0.92
BCL2-CR	0.59	0.45–0.73	1.451	0.32	0.94	0.54	0.35–0.73	1.451	0.33	0.91
BCL2-RL	0.55	0.33–0.77	2.148	0.32	1	0.56	0.27–0.84	1.655	0.5	0.8

Dx, diagnosis; PI, post-induction; CR, complete remission; RL, relapse; AUC, area under the ROC curve; OS, overall survival; CI, confidence interval.

Table S3. Dichotomized value of *BCL2* expression level with the area under the ROC curve for progression-free survival (PFS)

	PFS whole series					PFS in <70 years and intermediate cytogenetic risk				
**Variable**	**AUC**	**95% CI**	**Cutoff**	**Sensitivity**	**Specificity**	**AUC**	**95% CI**	**Cutoff**	**Sensitivity**	**Specificity**
BCL2-DX	0.56	0.45–0.67	0.8095	0.79	0.40	0.54	0.40–0.68	1.534	0.64	0.55
BCL2-PI	0.59	0.47–0.72	0.9932	0.47	0.76	0.58	0.42–0.75	0.9745	0.5	0.67
BCL2-CR	0.60	0.46–0.74	0.9661	0.49	0.77	0.54	0.35–0.72	1.427	0.28	0.875

Dx, diagnosis; PI, post-induction; CR, complete remission; RL, relapse; AUC, area under the ROC curve; OS, overall survival; CI, confidence interval.

Table S4. Univariate and multivariate progression-free survival analysis in the whole series at diagnosis (Dx)

	PROGRESSION FREE SURVIVAL Whole series							
	Univariate analysis				Multivariate analysis			
	Nº	HR	95% CI	P	Nº	HR	95% CI	P
**Age at Dx**	160	1.02	1.01–1.04	0.00076	138	1.023	1.01–1.04	<0.001
**ELN Risk**								
Low	150	1 (ref)			138			
Intermediate		1.20	0.71–2.00	0.50		1 (ref)		
High		1.71	1.02–2.86	0.04		1.056	0.62–1.81	0.842
***BCL2*-DX (continuous)**	147	1.09	0.93–1.28	0.30	138			
***BCL2*-DX (high*)**	147	1.63	1–2.66	0.05	138	1.589	0.96–2.62	0.07

ELN, European LeukemiaNet 2010; DX, diagnosis; * *BCL2* expression above AUC value (dichotomized); HR, hazard ratio; CI, confidence interval.

Supplementary Table S5. Univariate survival analysis.

	Overall Survival								Progression-Free Survival							
	Whole series				Intermediate cytogenetic risk and age <70 years				Whole series				Intermediate cytogenetic risk and age <70 years			
	Nº	HR	95% CI	P	Nº	HR	95% CI	P	Nº	HR	95% CI	P	Nº	HR	95% CI	P
**Age (continuous)**	176	1.04	1.02–1.05	0.000002	98	1.02	1–1.05	0.059	160	1.02	1.01-1.04	0.00076	91	1.01	0.99–1.04	0.22
**ELN risk**	160				93				150				87			
Low		1 (ref)								1 (ref)						
Intermediate		1.53	0.89–2.61	0.12						1.20	0.71–2.00	0.50				
High		2.10	1.22–3.62	0.008						1.71	1.02–2.86	0.04				
**BCL2- PI (continuous)**	91	1.45	1.01–2.07	0.046	52	1.63	1.04–2.57	0.035	91	1.39	0.99–1.97	0.06	52	1.48	0.97–2.25	0.069
**BCL2- PI (high)***	91	1.94	1.08–3.49	0.026	52	2.44	1.06–5.64	0.037	91	1.55	0.94–2.56	0.087	52	1.48	0.75–2.92	0.26
**BCL2-CR (continuous)**	67	1.83	1.14–2.95	0.012	41	1.54	0.85–2.77	0.15	67	1.63	1.07–2.49	0.022	41	1.35	0.81–2.28	0.25
**BCL2- CR (high)***	67	3.04	1.45–6.36	0.0033	41	2.71	1–7.33	0.049	67	2.06	1.11–3.85	0.023	41	1.97	0.81–4.79	0.14
**BCL2-RL (continuous)**	30	1.11	0.97–1.27	0.12	19	1.15	1–1.32	0.05	30	1.08	0.95–1.22	0.24	19	1.09	0.98–1.22	0.12
**BCL2- RL (high)***	30	1.22	0.52–2.85	0.65	19	1.72	0.59–4.99	0.32								

ELN, European LeukemiaNet; HR, hazard ratio; Nº, number of cases; *BCL2*-PI, *BCL2* levels at post-induction; *BCL2*-CR, BCL2 levels at complete remission; *BCL2*-RL, BCL2 levels at relapse; CI, Confidence Interval; P, p-value; * expression above AUC value (dichotomized)

Supplementary Figure S1. Correspondence between protein levels (**A**) via Western blot and mRNA levels (**B**) via QRT-PCR. Protein quantification (**C**) using ImageJ software. The images in (**A**) are taken from two different blots; the samples were derived from the same experiment and the blots were processed in parallel.

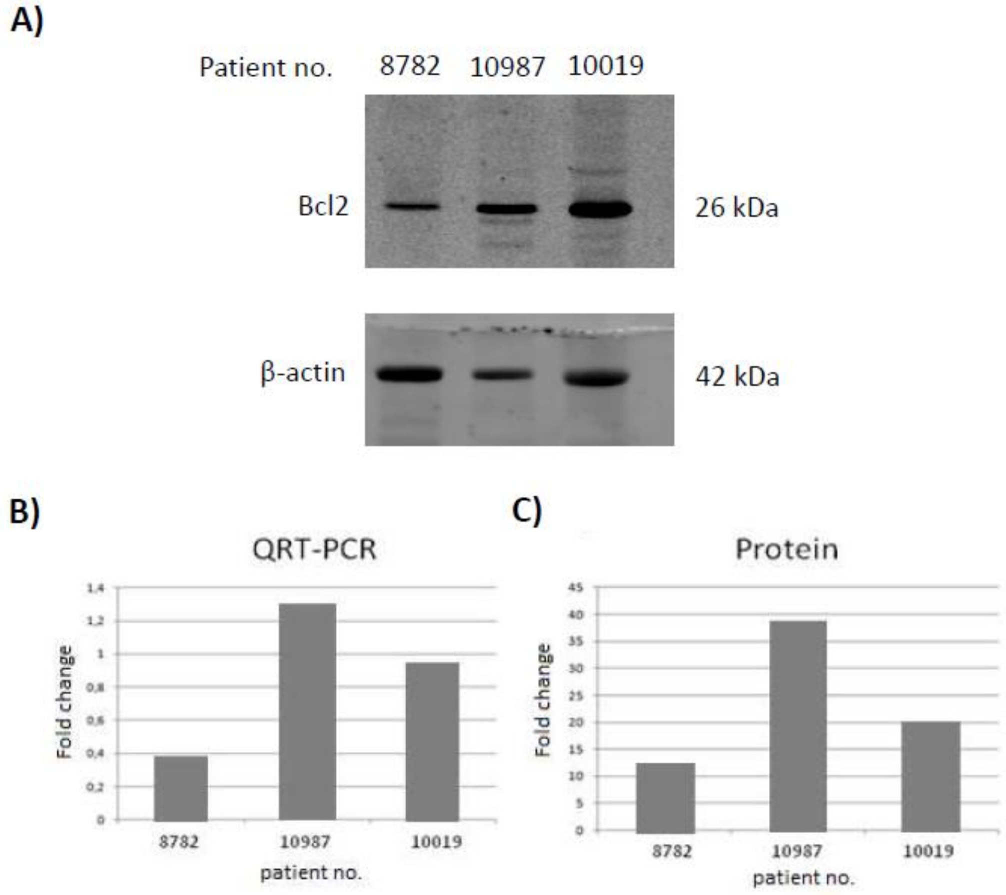

Supplementary Figure S2. Evolution of *BCL2* levels in 18 individual patients with follow up BCL2 values available at diagnosis (Dx), complete remission (CR) and relapse (RL)*.

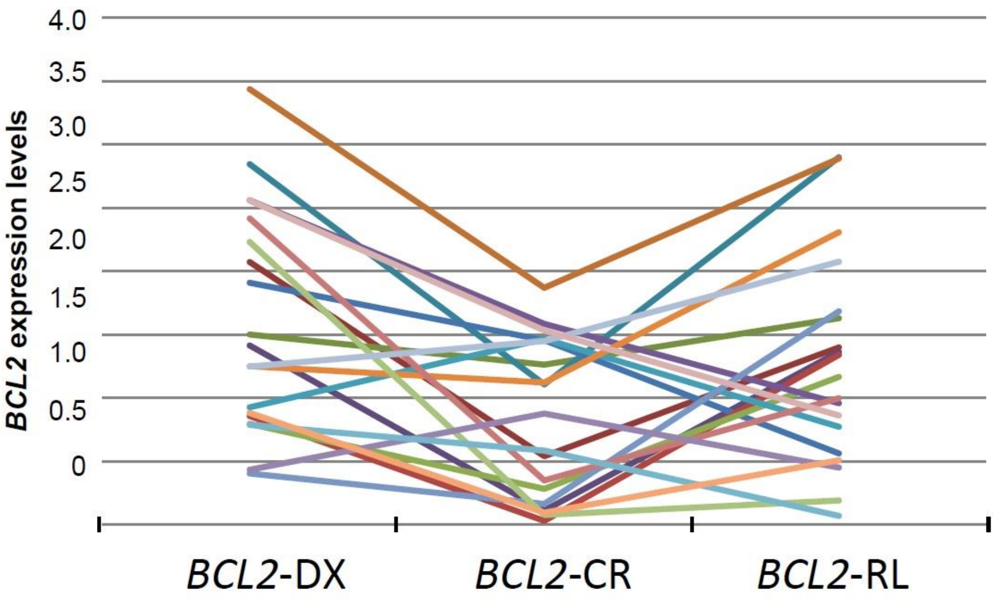

*Follow-up samples at three time points were only available for 23 patients and, of them, 18 patients were in CR at PI; therefore, we included only *BCL2*-CR levels.
